# Proteomics in Gastrointestinal Diseases of Companion Animals: Current State and Knowledge Gaps

**DOI:** 10.3390/ani16182814

**Published:** 2026-09-08

**Authors:** Jane Yu, Craig Ruaux

**Affiliations:** 1 Department of Veterinary Clinical Sciences, Jockey Club College of Veterinary Medicine and Life Sciences, City University of Hong Kong, Kowloon Tong, Hong Kong, China; 2 Department of Veterinary Clinical Sciences, College of Veterinary Medicine, University of Minnesota, Saint Paul, MN 55108, USA

**Keywords:** protein profiles, chronic enteropathy, biomarker discovery

## Abstract

Proteomics, the study of the structure and function of proteins, has been used to investigate candidate biomarkers and study the pathophysiology of various diseases. Biomarkers may be useful in diagnosing, monitoring and assessing treatment responses. This review provides an overview of proteomic techniques, describes the workflow and pipeline for biomarker discovery, and evaluates the current literature on proteomic studies in veterinary gastroenterology in dogs and cats. Current studies in this field are advancing our understanding of disease pathophysiology in conditions such as chronic enteropathy (CE). For biomarker development, proteomic research in veterinary gastroenterology is still in the early discovery phase. Substantial work on verification and validation is required before proteomic biomarkers can be reliably translated into clinical practice in companion animals.

## Simple Summary

Studying protein profiles is useful for investigating disease mechanisms and searching for biological markers. These biological markers can be used to diagnose diseases, monitor and predict treatment responses. This review provides an overview of the methods used to study proteins, outlines the process of discovering useful biological markers, and summarizes current research in dogs and cats with gastrointestinal diseases. Existing studies have improved our understanding of gastrointestinal diseases in dogs and cats. However, research in this area is still at an early stage. Potential biological markers have been explored but further studies are required to confirm their usefulness before they can be applied in routine clinical practice for dogs and cats.

## 1. Introduction

Gastrointestinal diseases are common clinical conditions encountered in small-animal practice. Diagnosis often involves a combination of clinical assessment, multiple diagnostic tests, and treatment trials [1,2]. Despite advances in diagnostic imaging, clinicopathological tests, histopathology, and ancillary techniques such as immunohistochemistry and molecular diagnostics, definitive diagnosis remains challenging in many cases [1,2]. Furthermore, histopathological findings often do not correlate with treatment response [2,3]. A significant amount of time and expense might be spent on treatment trials, and management, leading to owner fatigue and financial exhaustion. Minimally invasive diagnostic approaches using biomarkers have been explored in diagnosing CE [3]. Biomarkers may also be useful in disease monitoring, assessing severity, and assessing response to treatment [3]. In recent years, there has been a growing interest in the application of “omics” approaches in veterinary medicine to better understand disease pathophysiology and biomarker discovery [4,5]. Among these, proteomics, the large-scale study of proteins, including their function, structure, interactions, and cellular activities, has been increasingly explored [5,6]. However, when compared with human medicine, the development of proteomics in veterinary medicine remains in its infancy [5]. In humans, proteomic biomarkers are used to diagnose and monitor treatment response in enteropathies and represent novel, non-invasive, diagnostic and monitoring tools for inflammatory bowel disease (IBD) [4,7,8,9,10,11,12,13,14,15,16,17]. Several veterinary studies have been conducted to provide fundamental information and establish a foundation for future proteomic research in the field. These studies have investigated a variety of biological samples including serum, feces, and intestinal tissues, with the aim of studying the underlying pathophysiological process and investigating potential biomarkers for diagnosis, monitoring and treatment responses. This review provides an overview of the current use of proteomics in small-animal gastroenterology.

## 2. Methods

This narrative review provides current evidence on the application of proteomics in canine and feline gastrointestinal diseases, with a focus on chronic enteropathy. A structured search strategy was applied to improve reproducibility and minimize selection bias. The literature search was performed using the electronic databases PubMed, Web of Science and Scopus. Publications available up to May 2026 were considered. The search strategy included combinations of keywords including dog, dogs, canine, cat, cats, feline, proteomics, proteome, gastrointestinal, intestinal, gut, enteropathy, inflammatory bowel disease, and IBD.

For dogs, 158 records were identified across these databases. After removing 81 duplicate records, 77 unique records were retained. Title and abstract screening excluded 63 records that were not directly related to gastrointestinal disease, dogs, or proteomics, a study using dogs solely as experimentally induced disease models for human disease, and 5 review articles, resulting in 8 studies eligible for inclusion. For cats, 109 records were identified across databases. Following removal of 55 duplicate records, 54 unique records remained. Of these, 46 records were excluded because they were not directly related to gastrointestinal disease, cats, or proteomics. Two studies involving in vitro and experimentally induced disease models were excluded. Two studies were excluded because they focused primarily on parasite proteomics rather than host gastrointestinal proteomics. In addition, one review article and one published erratum were excluded. This screening process resulted in two feline studies being included.

Studies were eligible for inclusion if they investigated naturally occurring gastrointestinal disease in dogs or cats and employed a proteomic approach as a primary analytical methodology. Samples including tissues, serum, plasma, saliva, and feces were eligible. Experimental studies using dogs or cats solely as models for human disease, in vitro studies involving experimental infected models, amino acid profiling studies, studies unrelated to dogs or cats, conference abstracts and non-peer-reviewed studies were excluded. A manual review of the reference lists of included studies was performed to identify additional relevant literature.

Given the limited number of eligible studies and the heterogeneity in methodology across studies, formal risk-of-bias assessment was not conducted, and a quantitative meta-analysis was not possible. Instead, studies were critically evaluated based on disease population, methodology, biological and clinical relevance.

Ethical approval was not required because only previously published data were used in this review. No experimental procedures involving animals or sample handling were conducted.

## 3. Proteomics

The proteome is defined as the entire set of proteins that is expressed by a cell, while proteomics is the study of the structure and function of these proteins, and their interaction with each other [5,6]. By integrating protein abundance in different diseases, post-translational modifications, and protein interaction networks and pathways, proteomics can define and characterize dysregulated biological pathways, and provide insights into disease biology [5,18].

### 3.1. Workflow

The typical structure of a proteomic study involves several discrete steps. These include initial sample collection, protein extraction, and pre-analytical preparation, followed by identification and quantification of proteins using specific techniques [4]. Following sample collection, samples should be transported to the laboratory as soon as possible under controlled conditions, typically on ice or at 4 °C, depending on study design and sample type. Delays in transport and processing may lead to protein degradation or changes in protein abundances. Conditions during transportation should be standardized, and repeated freeze–thaw cycles should be avoided [19].

Various methods can be used to study the proteins within the sample, including the use of antibodies in immunoassays, electrophoretic assays, and mass spectrometry (MS) [5]. When analyzing complex samples, fractionation and separation of proteins from sample matrices may be required before characterization by MS to allow more accurate detection and quantification of proteins [5]. Statistical analysis and interpretation of the data are then carried out using techniques from the field of bioinformatics [4].

### 3.2. Biomarker Development

The discovery process for new proteomic biomarkers involves several distinct phases. The first phase of this process is exploratory, with screening for potential biomarkers using an untargeted approach using samples from a small group of subjects [4]. Samples including tissue, plasma, serum, urine or cell culture might be used [20]. During the discovery phase, mass spectrometry-based proteomic techniques are often used for large-scale protein profiling [4].

With the large number of distinct proteins present in most biological samples, it is typically advantageous to initially separate proteins based on physicochemical characteristics before conducting mass spectrometry analyses. Two-dimensional polyacrylamide gel electrophoresis (2DE) and liquid chromatography (LC) are the most commonly used techniques for protein separation prior to mass spectrometry (MS)-based techniques for protein quantification [4,21,22]. Two-dimensional electrophoresis (2DE) is often used for separation, detection and analysis of proteins [4]. The sample is processed in a gel matrix in which proteins move and form spots depending on the combination of their isoelectric point and molecular masses [5]. However, low-abundance, highly charged or very large proteins can be difficult to detect using this technique [5]. Using this method, recovery of proteins or peptides of interest by excision from the gel is needed before processing by MS [4]. This has several shortfalls, including the very large number of proteins present in typical biological samples, and the possibility that proteins of significant biological importance may be present in low total abundance.

Another widely used exploratory technique is liquid chromatography–mass spectrometry (LC-MS), which allows a more comprehensive study of proteins and better characterization of low-abundance proteins [4,6]. In LC-MS, a liquid chromatographic technique such as high-performance liquid chromatography is coupled with mass spectrometry to provide better sensitivity. This technique may also avoid sample loss and facilitate automation [4,5]. Mass spectrometry characterizes the analytes of interest by first converting analytes to an ionized state and measuring ions and (in the case of proteomics) fragment peptide ions produced during the process based on their molecular-mass-to-charge ratio [4,5,23]. As a result, information about protein structure is generated [5]. Electrospray ionization (ESI) and matrix-assisted laser desorption/ionization (MALDI) are the two major methods used for ionization of proteins for mass spectrometry processes [5]. Proteins in solution are used in ESI instruments while MALDI accepts analytes in a solid form [5]. MS is a useful tool for both identification and quantification of proteins; these data can then be used to probe biological properties and functional interactions [24]. Commonly, a combination of the electrophoretic technique (2DE) and LC-MS are used, where proteins are separated by electrophoresis followed by further fractionation by high-performance liquid chromatography (HPLC). Fractionated proteins are then analyzed by ESI mass spectrometry [5].

Other analytical techniques used in some proteomic methods include matrix-assisted laser desorption/ionization-time of flight (MALDI-TOF), tandem mass tag (TMT), sodium dodecyl sulfate-polyacrylamide gel electrophoresis (SDS-PAGE), surface-enhanced laser desorption ionization-time of flight (SELDI-TOF), isobaric tag for relative and absolute quantitation (ITRAQ), high-precision liquid chromatography–mass spectrometry (HPLC-MS), sequential window acquisition of all theoretical mass spectrometry (SWATH-MS), one-dimensional electrophoresis (1-DE) and in-gel digestion liquid chromatography–mass spectrometry (GeLC-MS) [6]. At the time of writing, the field of exploratory proteomics in veterinary research is dominated by 2DE and LC-MS or LC-MS approaches.

Proteomic approaches may be “bottom-up”, where complex protein mixtures are digested and cleaved to form peptides that are then analyzed by MS, or “top-down” where analysis of intact proteins takes place followed by the measurement of fragment ions by MS or gel-based methods [25]. Gel-free separation of proteins using isotope-tagging or labeling techniques can also be performed at a peptide level [4]. Each approach has its advantages and limitations. The “bottom-up” proteomic analysis is highly sensitive and allows identification of many proteins; however, information on post-translational modifications might be lost during the protein digestion process. The “top-down” approach allows preservation of information on isoforms and post-translational modification, but is more technically challenging [26].

**Figure 1:**
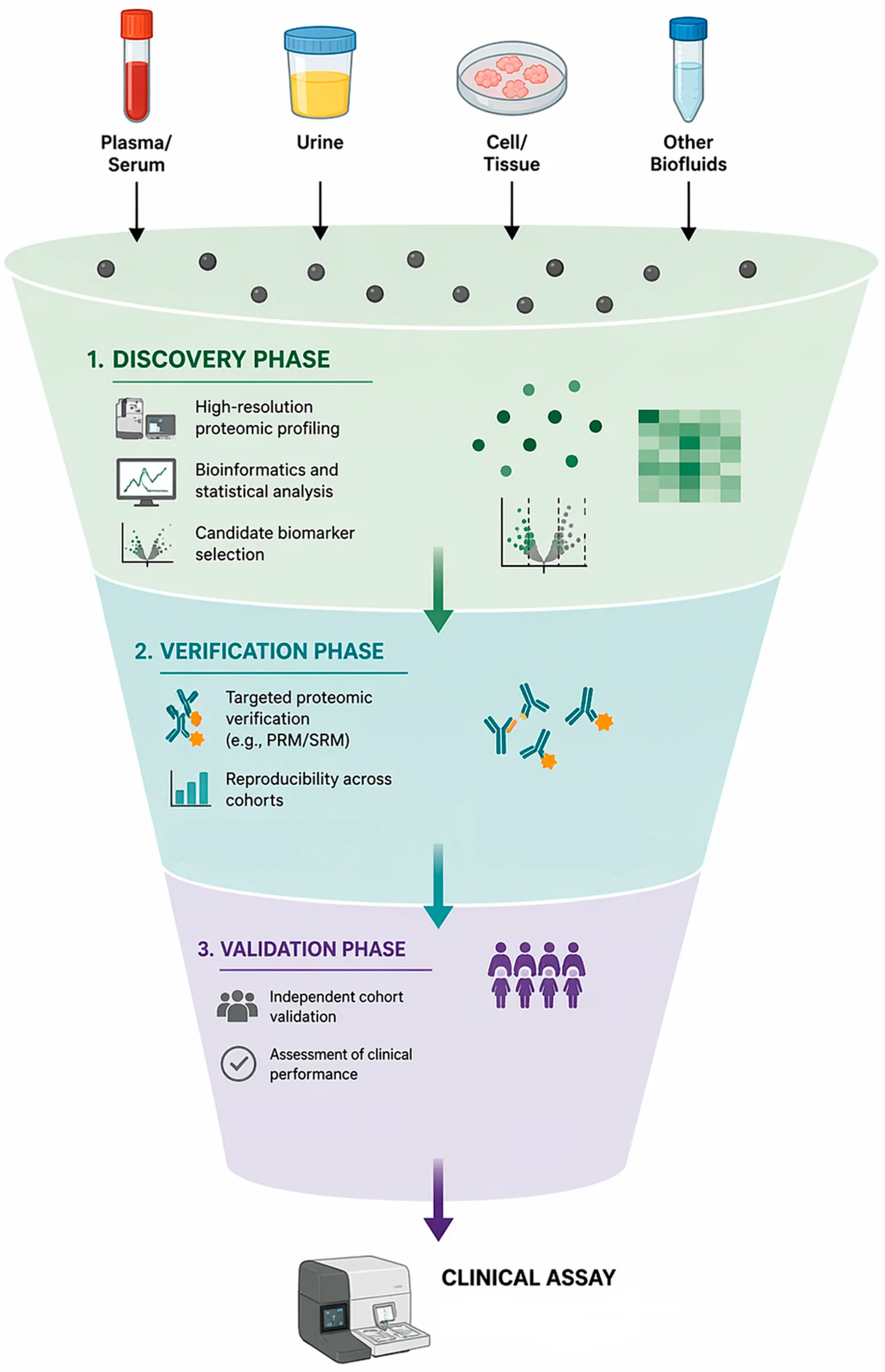
Biomarker development: Three stages are involved in the process of biomarker development. First, the discovery phase uses proteomic techniques to identify several candidate biomarkers. Samples might be plasma, serum, urine, tissue, cells or body fluids. Next, the verification stage involves using targeted techniques like parallel reaction monitoring (PRM) and selected reaction monitoring (SRM) to reproduce earlier results and further characterize these markers in other cohorts. In the final phase, candidate protein biomarkers undergo clinical validation to establish their clinical utility and assess their diagnostic or prognostic performance in certain populations. This image was generated using Microsoft Copilot (GPT-4o image generation) and subsequently reviewed and edited by the authors.

Following the initial discovery phase, the selected candidate protein biomarkers are assessed in the verification phase using targeted proteomic approaches, and are subsequently evaluated in a validation phase using a larger sample size to determine their clinical utility [4,21,22,27]. Methods commonly used for targeted approaches include protein microarrays, Western blotting, selected reaction monitoring and parallel reaction monitoring [4] (Figure 1).

An untargeted approach allows rapid identification and comprehensive coverage of many proteins. This approach is therefore particularly well suited for pathway exploration, biomarker discovery, and hypothesis generation [20]. However, this approach is only semi-quantitative, describing proteins in terms of relative abundance, and lacks precision and accuracy [20]. Detection of proteins of low abundance can be limited. Depending on the technique used, reproducibility may vary, and these approaches typically use expensive, complex analytical equipment requiring dedicated technical support [20]. In addition, cross-species identification can be challenging in animal studies due to incomplete database coverage of some species in protein structure databases [18]. As a result, the identification of some proteins may rely on apparent similarity with proteins of other species that have been studied. In comparison to untargeted approaches, however, the targeted approach is time and cost intensive to develop but offers precise quantification and high reproducibility [20,28]. As this approach focuses on proteins of interest, the target proteins must be known as the first step of assay development [20,28]. Challenges in the reproducibility of proteomic biomarker studies are common and may be affected by pre-analytical, analytical, and post-analytical factors. Pre-analytical variability has been discussed above. Analytical variability can arise from the use of different proteomic platforms and equipment, varying sample preparation methods, and differing protein detection workflows between research institutions. These factors may influence protein identification and quantification and contribute to differences in protein profiles reported between studies. Post-analytical variability may result from differences in bioinformatic and statistical methods, including data normalization, filtering criteria, and methods used for analyzing protein differential expressions. These factors limit direct comparison and reproducibility of findings across studies [29,30].

Currently, the application of proteomics in veterinary gastroenterology remains predominantly in the early discovery phase [5]. This discovery phase aims at identifying potential biomarkers by screening and analyzing entire protein profiles using an untargeted approach in carefully defined groups of patients, and comparing these patients to appropriately matched control samples [27]. Further verification and validation stages are then required, quantifying the potential biomarkers in larger groups of patients to assess their clinical value [27].

## 4. Proteomics in Small-Animal Medicine

In dogs, proteomic data have been reported in several areas other than gastrointestinal disease. Proteomic data are available from patients with Leishmaniasis, Ehrlichiosis, Staphylococcal disease, rabies, Babesiosis, B cell lymphoma, bladder and prostatic carcinoma, mammary carcinoma, osteosarcoma, oral neoplasia, hemangiosarcoma, myxomatous mitral valve disease, heartworm disease, dilated cardiomyopathy, cruciate disease, immune mediated anemia, immune mediated polyarthritis, osteoarthritis, chronic bronchitis, meningitis, cervical spondylomyelopathy, epilepsy, pyometra, chronic kidney disease, hepatobiliary diseases, diabetes mellitus, hypothyroidism and obesity [6,18,31,32,33,34,35,36,37,38,39,40,41,42,43,44,45,46,47,48,49]. A variety of sample types, including serum, plasma, urine and saliva, have been used in these studies [6,18,31,32,33,34,35,36,37,38,39,40,41,42,43,44,45,46,47,48,49,50]. In cats, proteomic data have been reported from animals with chronic kidney disease, mammary carcinoma, CE, pancreatic disease and osteoarthritis using serum, plasma and urine [5,51,52,53,54,55,56].

## 5. Gastrointestinal Diseases in Dogs

The utility of proteomics in gastrointestinal disease has not been widely explored in small animals. As in human medicine, most studies to date have aimed to explore disease pathophysiology and to identify novel proteomic biomarkers.

### 5.1. Acute Gastrointestinal Diseases

A small number of studies have explored proteomic data in dogs with acute gastrointestinal diseases. The fecal proteome has been investigated in eight dogs with acute uncomplicated diarrhea before and after treatment with dietary modification, probiotics, antiemetics or a combination of the above at different time points [57]. This study applied an untargeted, gel-based proteomic approach using 2DE coupled with mass spectrometry. Proteins are assessed semi-quantitatively based on gel spot intensity at different time points [57]. Four proteins, namely albumin, alkaline phosphatase, chymotrypsin-C-like protein, and immunoglobulins, were significantly different before and after treatment, and increased abundance was observed 14 days after the onset of disease [57]. These proteomic changes likely reflect pathologic changes in the gastrointestinal tract during acute diarrhea. Proteomic changes associated with intestinal epithelial damage, altered digestive function, activation of inflammation, and recovery mechanisms were observed in these dogs with acute diarrhea [57]. This study was limited by a small sample size and non-standardized treatment protocols, and etiologies were undetermined in the studied dogs [57]. Another study explored plasma protein profiles in 20 dogs with acute hemorrhagic diarrhea syndrome (AHDS) using an untargeted approach with relative quantification by label-free quantification (LFQ) LC–MS [58]. Several plasma proteins, including serpina3, lipopolysaccharide-binding protein, Ig-like domain-containing proteins, glyceraldehyde-3-phosphate dehydrogenase and serum amyloid A, were more abundant in dogs with AHDS compared to healthy controls, while decreased abundances of paraoxonase, selenoprotein, amine oxidases, and apolipoprotein C-IV were found in dogs with AHDS [58]. These changes in proteomic profiles are associated with inflammation, and these identified proteins could be potential biomarkers [58]. This study is limited by a small sample size and potential confounding factors due to differences in breed and body weight between groups [58]. The untargeted LFQ proteomic approach provides relative rather than absolute quantification [58]. While comparing dogs with AHDS and healthy controls provides useful baseline information, its clinical relevance is limited as AHDS is typically readily identifiable based on clinical presentation.

### 5.2. Parasitic Diseases

Proteomic profiles of small intestinal tissue homogenates in three dogs infected with *Toxocara canis* have been explored using a TMT-based, untargeted quantitative analysis method to assess the physiological and pathological effects of infection [59]. One hundred and ninety-eight proteins were found to be differentially expressed between infected dogs and controls; proteins involving development of infectious diseases, the immune system and signal transduction pathways were predominantly affected, suggesting that *Toxocara canis* may facilitate host invasion by suppressing immune responses and disrupting intestinal integrity [59]. The study also assessed metabolomic profiles in the intestinal tract of infected dogs; metabolites involving amino acid metabolism, bile secretion, tricarboxylic acid (TCA) cycle and glycolysis were altered in infected dogs. While this approach integrates proteomics and metabolomics into exploring pathways and mechanisms of host–parasite interaction, the strength of the study is limited by the very small sample size.

Overall, proteomic studies in dogs with acute gastrointestinal disease remain limited, and biomarker research is still largely at the discovery stage. While several candidate biomarkers have been identified, further validation is necessary before clinical application might be considered. To date, most proteomic studies have primarily focused on assessing the pathophysiological mechanisms of the underlying disease process. A summary of proteomic studies investigating acute gastrointestinal diseases in dogs is presented in Table 1.

### 5.3. Chronic Enteropathy

CE is common in dogs and can be classified into food-responsive enteropathy (FRE) and immunosuppressant-responsive enteropathy (IRE) based on treatment response [60,61]. Few studies have explored protein profiles in dogs with CE. The authors have investigated the serum proteomic profile in dogs with CE using an untargeted proteomic approach with an LC-MS method [18]. In addition, differences in serum proteomic profiles between dogs with differing forms of CE were investigated. Marked differences in protein abundances were found in 9 dogs with CE compared to 16 healthy controls, and protein profiles differed between subtypes of CE, for instance, FRE versus IRE [18]. Proteins involved in physiological pathways, such as reactive oxygen species generation, cytokine activation, acute phase response signaling, and lipid metabolism, were also different in dogs with CE compared to controls [18]. The study identified potential biomarkers, including transferrin receptor protein 1, gelsolin and fibronectin, that may differentiate between dogs with FRE and IRE [18]. This study provides valuable insights into the biology of CE, highlighting that distinct mechanisms may occur in different subtypes of the disease. However, these findings should be interpreted with caution given the small sample size and the absence of standardized diagnostic and treatment protocols [18].

The plasma proteomes of 10 dogs with histologically confirmed CE were compared to those of 10 healthy controls using an LFQ LC–MS method with an untargeted approach to identify and quantify differences in protein abundance between groups [62]. Several candidate biomarkers were detected [62]. Complement factor properdin was upregulated in dogs with CE and could be a potential biomarker for subclinical inflammation [62]. Hepatocyte growth factor activator was proposed to be a novel biomarker associated with decreased risk for CE in dogs [62]. Proteins such as inter-alpha-trypsin inhibitor heavy chain 4 and transcortin, and anti-inflammatory molecules such as apolipoprotein A-IV were significantly upregulated in remission. Transcortin and hepatocyte growth factor activator were proposed as biomarkers for remission in dogs with CE [62]. This study demonstrated that pathways associated with immune response and coagulation are altered in dogs with CE [62].

Fecal proteomic analysis using gel-based proteomics with 2DE followed by LC-MS, an untargeted approach, was performed in 12 dogs with CE and 7 healthy controls for protein profiling and biomarker identification [63]. Immunoglobulin J-chain isoform 1, a covalently bonded component of immunoglobulin A, was only present in feces of dogs with food-responsive diarrhea but not in healthy controls [63]. It is proposed that the presence of immunoglobulin J-chain isoform 1 in dogs with food-responsive diarrhea could be associated with increased immune activation from dysbiosis or mucosal damage [63]. Fecal proteomes of 11 dogs with CE and 14 healthy controls have also been investigated using an untargeted TMT-based quantitative proteomic approach by the same group [43]. Pancreatic-associated protein (REG3α), pancreatic M14 metallocarboxypeptidase proteins such as carboxypeptidase A1 and B, and several acute phase proteins including haptoglobin, lactotransferrin and transthyretin are significantly more abundant in feces of dogs with CE when compared to the healthy control group [43]. Alterations in bile acid metabolism suggesting dysbiosis were observed in further analysis [43]. A targeted approach was applied using SPARCL™ (Spatial Proximity Analyte Reagent Capture Luminescence) immunoassays to quantify fecal concentrations of pancreatitis-associated protein and canine haptoglobin, and results were correlated to the protein abundance found in TMT proteomics [43]. These studies identified a number of potential candidate fecal biomarkers for use in dogs with CE; however, further investigation is required to assess whether these biomarkers have diagnostic or prognostic utility.

In summary, proteomic studies suggest that CE in dogs is characterized by immune activation, dysbiosis and mucosal barrier damage, supported by alterations in bile acid metabolism. The condition is associated with dysregulation of pathways involving oxidative stress, cytokine activation, the acute phase response, coagulation, and lipid metabolism. These findings reveal a complex interplay between inflammation, gastrointestinal tract metabolism and the enteric microbiota. Most proteomic studies investigating canine CE are discovery based and have identified potential candidate biomarkers but lack subsequent validation. They share several main limitations. A major limitation applying to all studies is the small sample sizes. In addition, most studies focus on baseline comparisons between dogs with CE and healthy controls. While this information is valuable for generating foundational data, such comparisons have limited clinical utility, as the presence of gastrointestinal disease is typically evident in clinical practice with patients presenting with gastrointestinal signs. The more clinically relevant questions lie in differentiating between CE subtypes and distinguishing dogs with primary gastrointestinal disease from those with extra-gastrointestinal causes of chronic gastrointestinal signs. Additionally, most studies have a cross-sectional design with a lack of follow-up information. This lack of follow-up data limits our ability to determine if these potential biomarkers are useful for disease monitoring, treatment response or prognostication.

### 5.4. Protein-Losing Enteropathy

Protein-losing enteropathy (PLE) is a syndrome that involves plasma protein loss through the gastrointestinal tract; this can occur in various gastrointestinal diseases [64]. Etiologies of PLE include primary and secondary lymphangiectasia, lymphangitis, chronic inflammation, intestinal neoplasia and infectious and parasitic diseases [64,65]. Lymphangiectasia is commonly characterized by severe gastrointestinal signs with hypoalbuminemia and hypocholesterolemia [64,66]. The fecal proteome of dogs with lymphangiectasia has been investigated using an untargeted approach with 2DE and LC–MS [66]. In the same study, targeted biomarker quantification with ELISA and mass spectrometry-based assays was performed for selected proteins. Three proteins (Fc fragment of IgG-binding protein, transthyretin and proproteinase E) were found exclusively in fecal proteomics profiles in dogs with intestinal lymphangiectasia but not in clinically healthy dogs [66]. This study also suggested possible new diagnostic markers for dogs with lymphangiectasia, including serum and fecal C-reactive protein, bacterial lipopolysaccharide, cleaved cytokeratin 18, and zonulin [66].

## 6. Gastrointestinal Diseases in Cats

### Chronic Enteropathy

CE in cats can be categorized into FRE, idiopathic IBD (also known as IRE) and low-grade intestinal T-cell lymphoma (LGITL) [55,67]. Treatment trials with modified diets and histopathology using surgical or endoscopic biopsies are commonly required for definitive diagnosis [67]. In cats, the use of proteomics in gastrointestinal diseases has not been widely studied. One study investigated the intestinal mucosal proteome in cats with IBD and LGITL using 2D fluorescence difference gel electrophoresis and nanoflow LC-MS. Nine proteins were found to be differentially expressed between healthy cats and cats with IBD and LGITL during this discovery phase [56]. However, further verification with Western blot analysis did not confirm significant differential protein expression for the proteins that were assessed [56]. This study is limited by the small sample size and methodological constraints, including low-throughput gel-based techniques and the limited sensitivity and reliability of Western blot analysis [56].

In another study, the serum proteome in cats with CE was investigated using untargeted shotgun nanoLC–MS [55]. In this study, 26 proteins were significantly differentially abundant in cats with CE compared to healthy controls, and a potential candidate biomarker, thrombospondin-1, was upregulated in cats with CE [55]. Further analysis identified pathophysiological pathways involving epithelial damage and chronic inflammation [55]. Although thrombospondin-1 showed a marked increase in abundance in cats with CE in this study, this finding was not further validated, and additional investigation using targeted techniques is required [55]. In common with most veterinary discovery proteomic studies, this study is limited by the small sample size and non-standardized diagnostic and treatment protocol [55].

A summary of proteomic studies investigating chronic gastrointestinal diseases in dogs and cats is presented in Table 2.

## 7. Clinical Applications

Proteomics is a translational discipline that bridges research, laboratory work and clinical practice. While the generation, processing, and interpretation of proteomic data are most closely aligned with clinical pathology and laboratory diagnostics, the ultimate value of proteomics lies in its application in clinical veterinary practice. Through identifying alterations in protein profiles, dysregulated biological pathways and potential biomarkers associated with disease processes, proteomic analyses may provide useful information in diagnosis, prognostication and monitoring of treatment response [4,5]. Proteomic techniques are increasingly investigated in veterinary gastroenterology to study disease biology and investigate new biomarkers. These approaches have contributed to the discovery of many candidate biomarkers in various gastrointestinal diseases in dogs and cats. Despite this, clinical translation of these findings remains limited. Many biomarkers found from proteomic studies are still at the discovery phase and have not yet progressed to clinical use.

In small-animal gastroenterology, only a few studies, such as those investigating CE in dogs and cats and lymphangiectasia in dogs, have advanced slightly beyond an initial discovery phase [43,56,66]. Most studies remain exploratory, with only a small number incorporating quantitative analyses with small sample sizes [43,56,66]. This suggests a broader challenge in veterinary proteomics, where promising candidate biomarkers require extensive validation and verification in independent cohorts before clinical application [4]. The main steps involved are confirming reproducibility, and assessing diagnostic performance and robustness across other populations with larger sample sizes [4]. These steps are important to ensure consistent performance of the assay in a real-world setting. Therefore, although proteomics has the potential to advance veterinary diagnostics, substantial work is still needed to bridge the gap between biomarker discovery and practical clinical application.

With the currently available studies, differences in study design, methodologies, analytical approaches, and study populations, and lack of standardization between laboratories, make direct comparisons between studies challenging. This variability may contribute to inconsistent findings and represent an important barrier to biomarker development and clinical translation. In addition, accessibility of proteomic data in routine clinical practice is limited because proteomic analyses often require special equipment and bioinformatics expertise. Furthermore, the relatively limited availability of comprehensive proteomic reference databases in companion animals, compared with those available in human medicine, may further constrain protein identification, pathway analysis, and biomarker discovery, although these resources continue to expand [5].

Nevertheless, proteomic studies have provided valuable insights into the pathophysiology of gastrointestinal diseases by investigating profile profiles and altered pathways associated with inflammation, immune responses, epithelial barrier function, coagulation, oxidative stress, and other metabolic processes. These findings have improved our understanding of the underlying disease mechanisms, pathways and progression. Therefore, even when immediate clinical translation is not feasible, proteomics remains a valuable tool for advancing knowledge of disease biology [5].

## 8. Future Directions

Substantial work is needed before proteomics can be routinely applied in clinical veterinary practice. Future work might focus on the development of biomarker panels using multiple proteins rather than using individual proteins, as single proteins often have multiple biological roles and interact in a complex manner with multiple pathways. In addition, changes in a single protein may only be meaningful when there are alterations in other, unmeasured protein pathways. It is therefore unlikely that a single biomarker will provide sufficient diagnostic accuracy or discriminatory power to capture a disease or differentiate disease subtypes with complex pathophysiology. A panel of proteins integrated into a scoring system, algorithm, or artificial-intelligence-based analytical model is more likely to achieve clinical utility, as it can capture complex patterns and interactions among proteins.

Machine learning can be a valuable tool for data analysis and interpretation due to the large and complex datasets generated in proteomic studies [68,69]. Machine learning can be applied directly to mass spectral peak data or to proteins identified through proteomic analyses [70,71]. Its use has been reported for proteomic biomarker discovery and disease classification, particularly in cancer research, with potential applications in diagnosis, prognosis, and monitoring of treatment response [69]. Successful application of machine learning requires careful study design, including appropriate sample size, selection of suitable analytical methods and model validation [69]. Proteins identified through machine learning require further evaluation to determine their utility as clinically useful biomarkers and their relevance to disease processes [69].

A multi-omics approach integrating genomics, transcriptomics, metabolomics, and proteomics might provide a more comprehensive understanding of disease mechanisms from different perspectives, as these techniques complement proteomic data by providing additional biological information [72].

The gastrointestinal microbiome comprises a diverse community of organisms such as bacteria, archaea, fungi, protozoa, and viruses, and dysbiosis is seen in dogs and cats with CE [73,74]. Emerging approaches such as metaproteomics allow the detection of proteins expressed by the gut microbiome [75,76]. This further enables the study of their function, pathways and host–microbiome interactions [75,76]. Integrating host proteomic study with microbiome-based approaches such as metaproteomics, metagenomics, metatranscriptomics, and metabolomics might provide a comprehensive understanding of gastrointestinal disease pathogenesis and aid in identification of potential biomarkers [77].

Finally, to progress beyond the discovery phase, verification and validation phases with larger sample sizes and well-defined cohorts are necessary for further development of many biomarkers. This includes assessing biomarker performance in clinically relevant populations; for example, comparing dogs and cats with gastrointestinal signs due to primary gastrointestinal disease versus those presenting with similar clinical signs with extra-gastrointestinal conditions. Longitudinal studies evaluating proteomic changes over time and in response to treatment may improve understanding of disease progression and support the identification of biomarkers for disease monitoring and treatment response. In addition, verification of proposed biomarkers in independent cohorts from different institutions will be useful to confirm their reproducibility and robustness. Ultimately, rigorous validation and verification across diverse populations will be essential to bridge the gap between discovery and clinical implementation.

## 9. Conclusions

Proteomic studies in gastrointestinal diseases have revealed complex, interrelated pathophysiological pathways in conditions such as CE in companion animals, providing valuable insights into disease biology. For biomarker development, proteomic research in veterinary gastroenterology remains largely in the early discovery stage. The relatively few proteomic studies reported to date have identified some potential candidate biomarkers for diagnosis or assessment of clinically important diseases. However, extensive work, including verification and validation stages, is needed before proteomic biomarkers can be implemented in clinical practices in companion animals. Consequently, no proteomic biomarkers are currently ready for routine clinical application.

Most proteomic studies on gastrointestinal diseases in companion animals have focused on canine CE. Collectively, these studies suggest that CE is associated with immune activation, cytokine signaling, acute phase responses, oxidative stress, coagulation and altered lipid metabolism. These pathways suggest host–microbiome interactions and intestinal barrier dysfunction in disease pathogenesis. These findings highlight the value of proteomics for investigating disease mechanisms in addition to identifying candidate biomarkers.

This review provides a summary of the current state of proteomic research in veterinary gastroenterology in dogs and cats, outlines the methodologies used and their limitations, and highlights how these techniques may advance our understanding of disease pathophysiology, support biomarker discovery, and contribute to future clinical applications in companion animal medicine.

## Figures and Tables

**Table 1:** Summary of proteomic studies investigating acute gastrointestinal diseases in dogs.

Condition	Sample Type	Study Population	Proteomic Approach	Regulated Proteins/ Potential Biomarkers Identified	Proposed Mechanisms/Pathways	Reference
Acute uncomplicated diarrhea before and after treatment	Feces	8 dogs with acute diarrhea	Untargeted 2DE coupled with mass spectrometry	Albumin, alkaline phosphatase, chymotrypsin-C-like protein, immunoglobulins increased after treatment. No proposed biomarkers were reported.	Intestinal epithelial injury, altered digestive function, activation of inflammatory and recovery mechanisms	[57]
AHDS	Plasma	20 dogs with AHDS and healthy controls	Untargeted LFQ LC-MS	Increased serpina3 *, lipopolysaccharide-binding protein *, Ig-like domain-containing proteins *, glyceraldehyde-3-phosphate dehydrogenase *, serum amyloid A * and decreased paraoxonase, selenoproteins, amine oxidases, apolipoprotein C-IV found in dogs with AHDS	Changes in protein profiles are associated with inflammation and some are related to oncogenesis.	[58]
Toxocara canis infection	Small intestinal tissue	3 infected dogs and controls	Untargeted TMT-based quantitative proteomics (LC-MS)	198 differentially expressed proteins. No biomarkers were identified.	Immunosuppression, disruption of intestinal integrity, altered host defense mechanisms, signal transduction pathways	[59]

* Proposed candidate biomarkers reported by the original study.

**Table 2:** Summary of proteomic studies investigating chronic gastrointestinal diseases in dogs and cats.

Condition	Sample Type	Study Population	Proteomic Approach	Regulated Proteins/Potential Biomarkers Identified	Proposed Mechanisms/Pathways	Reference
Dogs						
CE	Serum	9 dogs with CE (including FRE and IRE) and 16 healthy controls	Untargeted LC-MS	Differential serum protein profiles between CE and healthy dogs and between FRE and IRE. Candidate biomarkers for distinguishing FRE from IRE included transferrin receptor protein 1 *, gelsolin *, and fibronectin *	Reactive oxygen species generation, cytokine activation, acute phase response signaling, and lipid metabolism.	[18]
CE	Plasma	10 dogs with histologically confirmed CE and 10 healthy controls	Untargeted LFQ LC-MS	Complement factor properdin * was upregulated and was a potential biomarker for subclinical inflammation. Hepatocyte growth factor activator * was associated with decreased risk of CE and was proposed to be a biomarker for remission. Inter-alpha-trypsin inhibitor heavy chain 4 and apolipoprotein A-IV were upregulated in remission. Transcortin * is a potential biomarker for remission.	Altered immune response and coagulation pathways.	[62]
Food responsive diarrhea	feces	12 dogs with CE and seven healthy controls	Untargeted gel-based proteomics (2DE coupled with LC-MS)	Immunoglobulin J-chain isoform 1 detected only in dogs with food responsive diarrhea only.	Increased mucosal immune activation associated with dysbiosis or intestinal mucosal injury.	[63]
CE	feces	11 dogs with CE and 14 healthy controls	Untargeted TMT-based quantitative proteomics; targeted validation by SPARCL™ immunoassays	Increased abundance of pancreatic-associated protein (REG3α *), carboxypeptidase A1, carboxypeptidase B, haptoglobin *, lactotransferrin *, and transthyretin * was found in dogs with CE. REG3α and haptoglobin were further quantified by targeted assays and results correlated with untargeted approach.	Alterations in bile acid metabolism suggested dysbiosis, acute phase and inflammatory responses.	[43]
Intestinal lymphangiectasia (PLE)	Feces	16 dogs with intestinal lymphangiectasia and seven dogs with other GI diseases	Untargeted 2DE coupled with LC-MS; targeted ELISA and MS-based assays	Fc fragment of IgG-binding protein, transthyretin, and proproteinase E were identified exclusively dogs with lymphangiectasia. Proposed diagnostic markers are serum and fecal C-reactive protein *, bacterial lipopolysaccharide *, cleaved cytokeratin 18 *, and zonulin *.	Intestinal barrier integrity, inflammation, bacterial translocation and protein loss associated with lymphatic disease.	[66]
Cats						
CE	Intestinal mucosa tissue	Six cats with IBD, eight with LGITL, and six healthy controls	2D fluorescence difference gel electrophoresis and nanoLC-MS	Nine proteins were differentially expressed during discovery analysis. Findings were not confirmed by Western blot validation. No biomarkers were identified.	No specific pathway was reported.	[55]
CE	Serum	10 cats with CE and 19 healthy controls	Untargeted shotgun nanoLC-MS proteomics	Twenty-six proteins were differentially abundant. Thrombospondin-1 was upregulated and proposed as a candidate biomarker.	Epithelial injury and chronic inflammatory pathways.	[56]

* Proposed candidate biomarkers reported by the original study.

## Data Availability

No new data were created or analyzed in this study. Data sharing is not applicable to this article.

## Abbreviations

The following abbreviations are used in this manuscript:

CE	Chronic enteropathy
IBD	Inflammatory bowel disease
MS	Mass spectrometry
2DE	Two-dimensional polyacrylamide gel electrophoresis
LC	Liquid chromatography
LC-MS	Liquid chromatography–mass spectrometry
ESI	Electrospray ionization
MALDI	Matrix-assisted laser desorption/ionization
HPLC	High-performance liquid chromatography
MALDI-TOF	Matrix-assisted laser desorption/ionization-time of flight
TMT	Tandem mass tag
SDS-PAGE	Sodium dodecyl sulfate-polyacrylamide gel electrophoresis
SELDI-TOF	Surface-enhanced laser desorption ionization-time of flight
ITRAQ	Isobaric tag for relative and absolute quantitation
HPLC-MS	High precision liquid chromatography-mass spectrometry
SWATH-MS	Sequential window acquisition of all theoretical mass spectrometry
1-DE	One-dimensional electrophoresis
GeLC-MS	In-gel digestion liquid chromatography-mass spectrometry
PRM	Parallel reaction monitoring
SRM	Selected reaction monitoring
AHDS	Acute hemorrhagic diarrhea syndrome
LFQ	Label-free quantification
FRE	Food-responsive enteropathy
IRE	Immunosuppressant-responsive enteropathy
SPARCL™	Spatial Proximity Analyte Reagent Capture Luminescence
PLE	Protein losing enteropathy
ELISA	Enzyme linked immunosorbent assay
LGITL	Low-grade intestinal T-cell lymphoma
